# Supplementary material for: Concentration of asset owners exposed to power sector stranded assets may trigger climate policy resistance
Source: Nat Commun. 2023 Oct 13;14:6442. doi: 10.1038/s41467-023-42031-w (PMC10576069; doi:10.1038/s41467-023-42031-w)
Supplement: Supplementary file 1 — Supplementary Information [file 41467_2023_42031_MOESM1_ESM.pdf]

Concentration of asset owners exposed to power sector stranded assets may trigger climate policy resistance

Inventory of supporting information

Supplementary methods

This section includes information on power plant standard lifetime assumptions, OCC assumptions, Asset Resolution data imputation, and Yahoo Finance data.

Supplementary results

This section includes additional results on the spatial distribution of stranded assets, sensitivity analyses, shareholder engagement, and further results.

Concentration of asset owners exposed to power sector stranded assets may trigger climate policy resistance

Supplementary information

Supplementary methods

Power plant standard lifetime assumptions. We assume that coal, gas, and oil plants have a standard lifetime of 50, 40, and 40 years, respectively (1; 2). Power plants' OCC may recover over shorter periods, in which case we would overestimate stranded assets. Thus, we alter power plant standard lifetimes in a sensitivity analysis (see below).

OCC assumptions. OCC cover a power plant's pre-construction, construction, and contingency costs but exclude interest during its construction – as if the plant was built overnight (3). OCC are used to evaluate and assess different power plant project options (4). OCC for the US, the EU, China, and India are taken from the IEA (5). OCC for Australia, Canada, Japan, Korea, Mexico, and Brazil are taken from the IEA and NEA (3).[1] OCC of other OECD countries are the average of OCC from the OECD countries mentioned and the EU. Likewise, OCC of other non-OECD countries are averages of OCC of China, India, and Brazil. All OCC used in the analysis are shown in Table 1.

Asset Resolution data imputation. For power plants with missing

[1]If multiple OCC estimations are provided for a country, we take their average. We exclude OCC of plants with carbon capture, utilization, and storage in this calculation as they greatly exceed OCC of conventional power plants and would distort the estimations upwards. For the OCC estimations of gas power plants in Australia, Canada, and Brazil, we consider both combined- and open-cycle gas turbine plants.

Table 1: Overnight capital costs per technology and country.

	Overnight capital costs [US$/kW]	
Country	Coal power plants	Gas/oil power plants
Australia	3095	812
Canada	-	906
EU	2000	1000
Japan	2419	1109
Korea	1151	973
Mexico	-	601
US	2100	1000
Other OECD countries	2153	914
Brazil	2189	847
China	800	560
India	1200	700
Other non-OECD countries	1396	702

installation year ($n = 26$), we use their decommissioning year and impute their installation year assuming the power plant lifetimes above. For power plants with missing information on both installation and decommissioning year (14 % of all plants), we assume the median installation year of power plants with the same technology located in the same country. For 64 plants, this imputation is not possible, since no other plant of the same technology exists in the same country. In such cases, we impute the installation year using the median installation year of power plants with the same technology located in OECD and non-OECD countries, respectively. We then proceed by imputing the decommissioning years of the power plants assuming the lifetimes above. Table 2 shows the fossil fuel power plant descriptive statistics after imputing missing values.

In the base year of the analysis, namely 2020, the Asset Resolution data

Table 2: Fossil fuel power plant descriptive statistics.

	mean	std	min	25 %	50 %	75 %	max
Installation year	2001	8	1924	1996	2000	2005	2036
Decommissioning year	2041	9	2020	2036	2039	2045	2085
Capacity [MW]	72.35	194.59	0.01	1.80	2.80	18.00	6000.00

covers only between 62 % and 86 % of the global fossil power plant capacity outlined in the IEA data depending on the fuel type and region (see Table 3).

Table 3: Shares of power plant capacity in IEA data covered by Asset Resolution data.

Region	Coal	Gas	Oil
ASIAPAC-CN-IN-JP	1.00	0.98	0.73
Africa	0.97	0.86	0.81
BR	0.75	1.06	0.90
CN	0.78	0.72	0.49
CSAM-BR	1.06	0.98	0.74
EUR	0.83	0.83	0.42
EURASIA-RU	0.77	0.78	0.09
IN	1.01	1.08	0.54
JP	0.86	0.81	0.37
ME	1.06	0.91	0.85
NAM-US	0.99	1.02	0.45
RU	0.50	0.53	0.16
US	0.88	0.88	0.32
World	0.84	0.86	0.62

Region abbreviations are as described in Figure 1.

To avoid underestimating asset stranding to due this data gap, we impute the capacity that is missing in the base year. More precisely, we construct power plants for each region and fuel type with the median age and capacity of the respective region and fuel type. For the constructed power plants, we assume the lifetimes above. We fill the data gap in the Asset Resolution

data for 2020 with these constructed power plants. For instance, for Brazil, we construct 8 coal power plants with a capacity of 131 MW aged 10 years. Since the lifetime of a coal power plant is assumed to be 50 years in the baseline analysis, these constructed power plants retire in our imputed Asset Resolution data in 2050. We assign constructed power plants to an unknown asset owner. In our stranding method, we strand power plants owned by such an unknown asset owner first if it has the same age as a power plant owned by a known owner. Constructing these power plants is thus important to identify and strand plants, which are above the median age (for a given region and technology) and would not be stranded otherwise due to the data gap. Stranded assets owned by unknown asset owners are excluded in the figures in the main text.

Finally, if the equity share between direct and parent owner is missing ($n = 9$), we manually search for the parent owner, which may be the major shareholder, on the direct owner's website and assume an equity share equal to one. If the equity shares of a direct owner do not add up to one ($n = 138$ or 2 % of direct owners owning stranded assets and $n = 194$ or 1 % of direct owners owning alternative energy assets), we scale up the equity shares provided in the data proportionately so that they sum up to one. For 504 direct and parent owners, we impute missing information on headquarter location by manually searching through their respective websites.

Yahoo Finance data. Table 4 shows descriptive statistics of the financial variables retrieved from Yahoo (6):

Table 4: Descriptive statistics of parent owner financial variables.

	mean	std	min	25 %	50 %	75 %	max
Shares outstanding [Mio.]	$7.7 \cdot 10^3$	$1.0 \cdot 10^5$	1.3	130.0	415.6	$1.3 \cdot 10^3$	$1.9 \cdot 10^6$
Market capitalization [Mio. US$]	$2.9 \cdot 10^4$	$6.1 \cdot 10^4$	5.6	$2.3 \cdot 10^3$	$8.4 \cdot 10^3$	$3.0 \cdot 10^4$	$6.8 \cdot 10^5$
Total equity [bn. US$]	17.8	38.7	-2.0	1.9	6.2	17.8	514.9

Supplementary results

Spatial distribution of stranded assets. The aggregation of stranded assets at the regional level potentially covers up within-region heterogeneity. Figure 9 shows stranded assets globally using the geolocation of prematurely retired power plants.

Figure 9: Spatial distribution of stranded assets between 2021 and 2050.

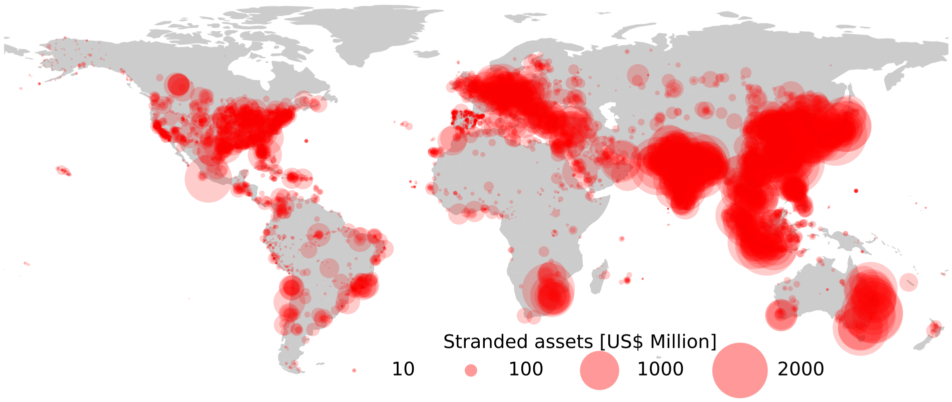

Each dot represents a stranded power plant including its geolocation. The size of the dot represents the monetary value of the stranded asset.

Sensitivity analyses. The quantification of stranded assets depends on

assumptions on interest rates and power plant lifetimes as shown in Table 5. Higher interest rates and lower power plant lifetimes decrease stranded assets.

Table 5: Global stranded assets [US$ Million] for different interest rates and power plant standard lifetimes.

Fossil fuel plant type	Plant standard lifetime [years]	Interest rate [%] 5	3	7
Coal	50	413983.0	506584.0	347448.5
	40	276876.8	339945.7	231023.9
	30	141912.0	176999.4	116350.6
	25	72755.3	90246.7	59812.4
Oil	40	15056.7	16607.5	13789.6
	30	2602.8	2871.6	2375.6
	25	324.4	361.4	292.4
	20	6.3	6.4	6.3
Gas	40	71158.6	77878.3	65889.1
	30	21196.6	22339.2	20151.4
	25	6503.4	6903.5	6139.5
	20	1767.7	1867.2	1675.7

Further, the stranded assets quantification depends on which power plant is stranded first, whenever the operating capacity exceeds the climate-compatible capacity. In our baseline analysis we strand the oldest power plants in a given region first. This approach aims at minimizing unrecovered capital costs and thus stranded assets. One could argue, however, that it is more realistic to strand power plants in the richest or poorest country in a given region first. We implement this alternative approach and rank power plants to be stranded in a given region by GDP per capita [current US$] in 2020[2] in the country the plant is located. The plant's age remains only relevant in case several plants are located in the same country. Results show that stranded assets increase to

[2]Data retrieved from IMF (7) and United Nations Statistics Division (8).

US$657 (632) billion if plants located in poor (rich) countries in a given region are stranded first.

Shareholder engagement. Shareholders may engage with listed asset owners exposed to stranded assets, e.g. via climate initiatives, which support asset owners aiming at climate targets. For instance, "Climate Action 100+" is an investor-led initiative with the goal to assist asset owners in their transition to net-zero emissions by active engagement (9). "Science Based Targets initiative" shows asset owners how quickly they need to reduce their emissions to reach net-zero targets and provides technical assistance in this process (10). A small subset of listed parent owners are members in these climate initiatives and together they own 8.5 % of stranded assets.

Further results.

Figure 10 shows the distribution of listed parent owners' stranded assets and total equity.

Table 6 shows asset owner names of the placeholders in Figures 3 and 4.

Figure 10: Stranded assets and total equity of listed parent owners headquartered in OECD and non-OECD countries.

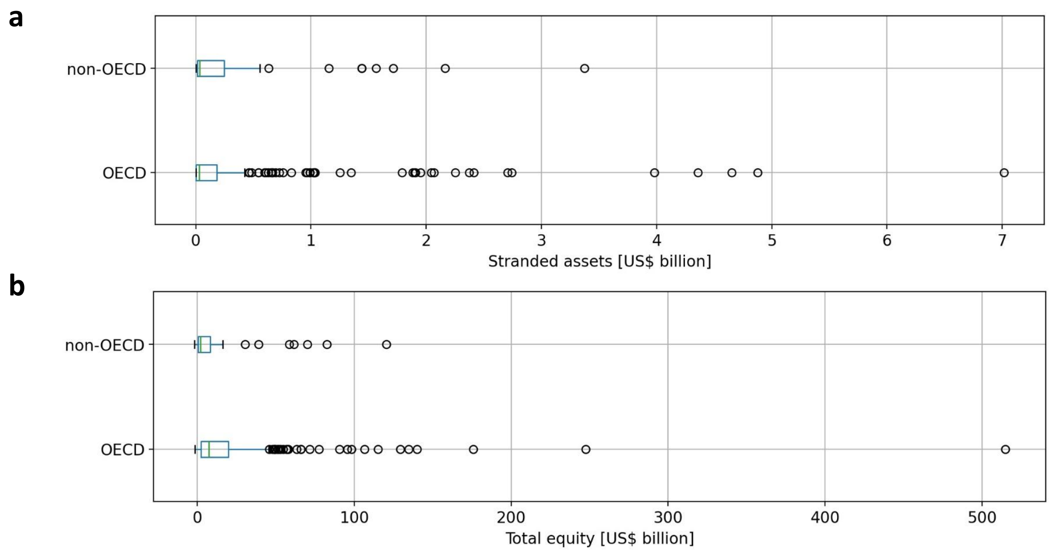

Panel a: Stranded assets of listed parent owners. Panel b: Total equity of listed parent owners. In each boxplot the box extends from the first to the third data quartile. In the box, the median of data is represented by a vertical line. Whiskers extent the box by a factor of 1.5 of the inter-quartile range and flier points exceed those whiskers.

Table 6: Asset owner names of placeholders in Fig. 3 and 4.

Fig.	Placeholder	Asset owner name
3 (a)	A	Huaneng Power International Inc
	B	Datang International Power Generation
	C	China Energy Investment Corp Ltd
	D	China Huaneng Group Co Ltd
	E	Huadian Power International Co
	F	China Datang Corp
	G	Shanghai Waigaoqiao Power Generation Co Ltd
	H	Zhejiang Beilun Power Generation Co Ltd
	I	Guangdong Energy Group Co Ltd
	J	China Huadian Corp Ltd
3 (b)	A	NTPC Ltd
	B	Mahagenco

Table 6 – continued from previous page

Fig.	Placeholder	Asset owner name
	C	Rajasthan Rajya Vidyut Utpadan Nigam Ltd
	D	Damodar Valley Corp
	E	Uttar Pradesh Rajya Vidyut Utpadan Nigam Ltd
	F	Tamil Nadu Generation & Distribution Corp Ltd
	G	Telangana State Power Generation Corp Ltd
	H	Adani Power Ltd
	I	The West Bengal Power Development Corp Ltd
	J	The Tata Power Co Ltd
3 (c)	A	Luminant Holding Co LLC
	B	Vistra Corp
	C	Calpine Corp
	D	NRG Energy Inc
	E	Duke Energy Carolinas LLC
	F	Florida Power & Light Co
	G	Santee Cooper
	H	Alabama Power Co
	I	Duke Energy Florida LLC
	J	Xcel Energy Inc
3 (d)	A	JERA Co Inc
	B	Tohoku Electric Power Co Inc
	C	Kyushu Electric Power Co Inc
	D	Hokuriku Electric Power Co
	E	Chugoku Electric Power Co Inc
	F	The Kansai Electric Power Co Inc
	G	Electric Power Development Co
	H	Hokkaido Electric Power Co Inc
	I	Kobelco Power Kobe Inc
	J	Nippon Steel Corp
4 (a)	A	NTPC Ltd
	B	Perusahaan Perseroan Persero PT

Table 6 – continued from previous page

Fig.	Placeholder	Asset owner name
	C	Huaneng Power International Inc
	D	Eskom Holdings SOC Ltd
	E	JERA Co Inc
	F	Taiwan Power Co
	G	Korea South-East Power Co Ltd
4 (b)	A	People's Republic Of China
	B	Republic Of India
	C	Republic Of Korea
	D	Republic Of Indonesia
	E	Republic Of South Africa
	F	Socialist Republic Of Vietnam
	G	Chubu Electric Power Co Inc

References

[1] Cui, R. Y. *et al.* Quantifying operational lifetimes for coal power plants under the Paris goals. *Nature Communications* **10**, 1–9 (2019).

[2] Tong, D. *et al.* Committed emissions from existing energy infrastructure jeopardize 1.5 °C climate target. *Nature* **572**, 373–377 (2019).

[3] IEA and NEA. Projected costs of generating electricity. OECD Publishing, Paris (2020).

[4] Koomey, J. & Hultman, N. E. A reactor-level analysis of busbar costs for US nuclear plants, 1970-2005. *Energy Policy* **35**, 5630–5642 (2007).

[5] IEA. World Energy Outlook 2021. OECD Publishing, Paris (2021).

[6] Yahoo. Yahoo Finance. https://finance.yahoo.com/ (2022). Accessed: 2022-07-08.

[7] IMF. World Economic Outlook Database. https://www.imf.org/en/Publications/WEO/weo-database/2020/October (2023). Accessed: 2023-03-14.

[8] United Nations Statistics Division. National accounts estimates of main aggregates. https://unstats.un.org/unsd/snaama (2030). Accessed: 2023-03-14.

[9] Climate Action 100+. Global investors driving business transition. https://www.climateaction100.org/ (2022). Accessed: 2022-01-19.

[10] Science Based Targets initiative. Ambitious corporate climate action. https://sciencebasedtargets.org/ (2022). Accessed: 2022-01-19.
